# Efficacy of prednisone 1–4 mg/day in patients with rheumatoid arthritis: a randomised, double-blind, placebo controlled withdrawal clinical trial

**DOI:** 10.1136/ard.2008.095539

**Published:** 2008-12-11

**Authors:** T Pincus, C J Swearingen, G Luta, T Sokka

**Affiliations:** 1 New York University Hospital for Joint Diseases, New York, NY, USA; 2 Medical University of South Carolina, Charleston, SC, USA; 3 Department of Biostatistics, Bioinformatics and Biomathematics, Georgetown University Medical Center, Washington, DC, USA; 4 Arkisto/Tutkijat, Jyväskylä Central Hospital, Jyväskylä, Finland

## Abstract

**Objective:**

A randomised double-blind placebo controlled withdrawal clinical trial of prednisone versus placebo in patients with rheumatoid arthritis (RA), treated in usual clinical care with 1–4 mg/day prednisone, withdrawn to the same dose of 1 mg prednisone or identical placebo tablets.

**Methods:**

All patients were from one academic setting and all trial visits were conducted in usual clinical care. Patients were taking stable doses of 1–4 mg prednisone with stable clinical status, documented quantitatively by patient questionnaire scores. The protocol included three phases: (1) equivalence: 1–4 study prednisone 1 mg tablets taken for 12 weeks to ascertain their efficacy compared with the patient’s usual tablets before randomisation; (2) transfer: substitution of a 1 mg prednisone or identical placebo tablet every 4 weeks (over 0–12 weeks) to the same number as baseline prednisone; (3) comparison: observation over 24 subsequent weeks taking the same number of either placebo or prednisone tablets as at baseline. The primary outcome was withdrawal due to patient-reported lack of efficacy versus continuation in the trial for 24 weeks.

**Results:**

Thirty-one patients were randomised, 15 to prednisone and 16 to placebo, with three administrative discontinuations. In “intent-to-treat” analyses, 3/15 prednisone and 11/16 placebo participants withdrew (p = 0.03). Among participants eligible for the primary outcome, 3/13 prednisone and 11/15 placebo participants withdrew for lack of efficacy (p = 0.02). No meaningful adverse events were reported, as anticipated.

**Conclusion:**

Efficacy of 1–4 mg prednisone was documented. Evidence of statistically significant differences with only 31 patients may suggest a robust treatment effect.

The use of glucocorticoids in the treatment of rheumatoid arthritis (RA) has evoked controversy for more than half a century.^1^ ^2^ ^3^ ^4^ ^5^ Disease modification was documented during the 1950s,^6^ but toxicities of long-term glucocorticoids in pharmacological doses of prednisone or prednisolone of 10 mg/day or more, as was the clinical practice in the 1950s,^7^ were inevitable. Therefore, from the 1950s through the 1980s, systemic glucocorticoids were recommended in RA only as “bridging therapy” while awaiting anticipated benefits of disease-modifying antirheumatic drugs (DMARDs), or for acute severe disease flares or life-threatening vasculitis.

A reassessment began during the 1980s, based on recognition of severe long-term outcomes of RA^8^ ^9^ and clinical experience indicating relatively limited toxicity associated with low doses of glucocorticoids. An open study,^10^ a 24-week non-blinded clinical trial^11^ and recent double-blind clinical trials^12^ ^13^ ^14^ ^15^ ^16^ ^17^ have recognised clinical benefit, including “disease-modifying” properties, of low-dose prednisone in slowing radiographic progression, confirmed in meta-analyses.^18^ ^19^ Reports indicating disease modification even with low doses of prednisone or prednisolone of 5–7.5 mg/day^12^ ^16^ ^17^ are of particular interest, as doses of 10 mg/day are associated with adverse outcomes^20^ including bone loss^21^ and higher mortality rates.^22^ ^23^

Prednisone or prednisolone for RA generally is initiated with a dose of 10–20 mg/day and maintained at levels of 5 mg/day or more. The medical literature includes varying criteria for “low-dose” prednisone, generally 5 mg or 10 mg/day. A few clinicians, including the senior author, have treated most patients over the last decade with an initial dose of 3 mg/day.

The efficacy of prednisone in doses of <5 mg/day has not been established in patients with RA, and rheumatologists continue to disagree on the use of glucocorticoids. A double-blind clinical trial to analyse the efficacy of <5 mg/day prednisone would therefore appear desirable. A large multicentre prospective randomised double-blind clinical trial in patients with no previous glucocorticoid therapy, to be taken with their usual RA treatment, might appear ideal. However, resources for such a multicentre clinical trial have not been available. Therefore, with partial support from the United States Arthritis Foundation, we performed a single-centre withdrawal trial of prednisone <5 mg/day in the course of usual care.

## Methods

### Patients

All patients were recruited from one academic clinical care setting at Vanderbilt University and all clinical trial visits were conducted during usual clinical care. Most patients with RA in this clinical setting have been treated with long-term prednisone 1–5 mg/day, with a usual initial dose of 3 mg/day since the mid-1990s. Clinical efficacy without severe toxicity has been observed,^24^ although almost all patients are also treated with methotrexate so the specific efficacy of prednisone could not be analysed without a clinical trial.

### Withdrawal clinical trial protocol

Patients with stable clinical status who were taking stable doses of prednisone 1–4 mg/day in 1 mg tablets or one 5 mg tablet per day (although no patients taking 5 mg were actually enrolled in the trial) over the previous 12 weeks were invited to participate in a randomised double-blind placebo controlled prednisone withdrawal clinical trial. All participants gave informed consent to participate. The trial was approved by the Institutional Review Board of Vanderbilt University, and supported in part by the United States Arthritis Foundation.

The trial was designed to be broadly inclusive with few exclusion criteria. Inclusion criteria were: age at least 18 years; met American Rheumatism Association (ARA) criteria for RA;^25^ had been taking a stable dose of 1–5 mg/day prednisone for at least 12 weeks with no anticipated dose change. Stable clinical status was documented by an absolute change of less than 3 units from 12 weeks earlier in routine assessment of patient index data 3 (RAPID3), an index of the three patient-reported outcomes, on a multidimensional health assessment questionnaire (MDHAQ)^26^ for physical function, pain and global estimate of status, each scored 0–10, total 0–30,^27^ completed by all patients at all visits as a component of the infrastructure of standard care.^28^

Exclusion criteria were relatively few: no prednisone therapy; prednisone dose >5 mg/day; improving or worsening clinical status; anticipation of joint replacement or other elective surgery; uncontrolled hypertension, diabetes or other comorbidities; severe fibromyalgia; inability to complete English language questionnaires; and pregnancy or nursing.

The protocol included three phases:

- Equivalence: all participants were given a 12-week (84-day; actually 100 days to ensure availability) supply of “study prednisone” tablets to take at the same dose as at baseline before entry into the clinical trial. These tablets were taken in lieu of the patients’ usual prednisone obtained at their own pharmacies to ascertain similar efficacy of the study prednisone to the usual prednisone.
- Transfer: participants who reported “equivalence” over the 12-week period were assigned randomly to be “transferred” at a rate of a single 1 mg tablet per 4 weeks over the next 0–12 weeks from study prednisone tablets to either 1 mg prednisone or identical placebo tablets (table 1). The gradual transfer was performed to avoid abrupt reduction of prednisone usage in subjects randomised for transfer to placebo.
- Comparison: participants were maintained over 24 weeks following the “transfer” phase on the same number of either 1 mg prednisone or identical placebo tablets as at baseline.

**Table 1:** Plan to “transfer” patients from low-dose prednisone tablets to study prednisone or placebo tablets

Dose	Medication	Week of “transfer” phase				
		Week 0	Week 4	Week 8	Week 12	Week 16*
1 mg	Bottle A (prednisone)	0				
	Bottle B (unknown)	1				
2 mg	Bottle A (prednisone)	1	0			
	Bottle B (unknown)	1	2			
3 mg	Bottle A (prednisone)	2	1	0		
	Bottle B (unknown)	1	2	3		
4 mg	Bottle A (prednisone)	3	2	1	0	
	Bottle B (unknown)	1	2	3	4	
5 mg	Bottle A (prednisone)	4	3	2	1	0
	Bottle B (unknown)	1	2	3	4	5

Each participant was given an individual schedule outlining specific dates to make changes in the number of tablets to be taken from bottle A and bottle B.

*No patients taking 5 mg at baseline were enrolled in the study.

Each visit included assessment and recording of weight and blood pressure; completion of an MDHAQ by the patient and scoring of RAPID3 by the rheumatologist; and laboratory tests of erythrocyte sedimentation rate (ESR), C-reactive protein (CRP), liver function and haematological status to monitor possible adverse events of prednisone or concomitant methotrexate or other medications.^29^

### Prednisone and placebo tablets

Prednisone 1 mg tablets and identical placebo tablets were purchased from Apotex Inc, Toronto, Canada. Tablets were packaged in bottles containing 100 tablets each. The bottles were relabelled at the trial site with two types of labels: “Bottle A”, known to be prednisone, and “Bottle B” which contained “unknown” tablets (either prednisone or placebo). The appropriate number of Bottle A study prednisone tablets in bottles of 100 tablets (according to the daily dose at baseline (1–5 mg/day; no patient enrolled took 5 mg/day)) was given to each participant for a 100-day supply during the 12-week (84-day) “equivalence” phase.

Packets were prepared for visits 2, 3 and 4 based on the participant’s daily baseline dose (1–5 mg/day), according to a randomisation scheme in groups of 4 (2 prednisone and 2 placebo) for each dose. Packets for the entire study were prepared for 68 possible participants, 8 each for prednisone dose levels of 1, 2, 4 or 5 mg/day and 36 for 3 mg/day, taken by the majority of patients before the trial. Study packets included the appropriate number of “Bottle A” bottles of 100 1 mg prednisone tablets and “Bottle B” bottles of 1 mg prednisone or identical placebo tablets (table 1).

### Study visits

Visit 1 included an explanation of the trial and completion of informed consent. Participants were given a 12-week supply (with tablets for 16 extra days) of “Bottle A” 1 mg study prednisone tablets to take instead of their usual daily prednisone dosage for 12 weeks. This phase was designed to establish whether or not “equivalence” of the same dose of study prednisone to the patient’s usual prednisone tablets could be seen.

Visit 2 occurred 12 weeks later. Participants who reported “equivalence” of study prednisone to their usual prednisone dose over the 12 weeks and elected to continue in the study were randomised to either 1 mg prednisone or identical placebo tablets for the “transfer” phase. Each participant received a specific written schedule with specific dates every 4 weeks to reduce by one the number of tablets to be taken from “Bottle A” (of 1 mg prednisone tablets) and to increase by one the number of “Bottle B” (of unknown study tablets, either 1 mg prednisone or identical placebo tablets) over a 12-week period. Substitution of one “Bottle B” tablet for one “Bottle A” tablet occurred at 0, 4, 8, 12 and 16 weeks for 1, 2, 3, 4 and 5 mg baseline dose, respectively (no enrolled participant was taking 5 mg/day). Therefore, at the end of the 12-week period, each participant was taking only “Bottle B” study medication. The gradual tapering was designed to avoid an abrupt discontinuation of prednisone which might favour prednisone.

Visit 3 occurred 4–12 weeks after visit 2 (or was omitted for participants with a baseline dose of 1 mg/day). All participants whose baseline daily dose was 2–4 mg prednisone then started taking only unknown Bottle B prednisone or placebo and began the “comparison” phase.

Visit 4 occurred 12 weeks after visit 3. Participants completed the usual MDHAQ and the trial status was reviewed with the investigator.

Visit 5 occurred 12 weeks after visit 4, at least 24 weeks after participants had completed the “transfer” phase. The participants completed the final usual MDHAQ and prednisone was reinstated at the pretrial dose.

### Clinical trial outcomes

The predetermined primary outcome was withdrawal (“drop-out”) after visit 2 due to perceived lack of efficacy of study tablets (prednisone or placebo), ie, during the “transfer” or “comparison” phases of the trial, versus remaining in the trial until completion of the 24-week “comparison” period. Secondary outcomes included a change in any of the three RA Core Data Set variables^30^ found on the MDHAQ (as well as the HAQ) for physical function, pain and global estimate, all scored 0–10, and RAPID3 0–30 composite scores. Weight, systolic and diastolic blood pressure and laboratory tests of ESR, CRP, haematology and liver profiles were recorded at each visit and analysed as indicators of possible adverse events.

### Data management and statistical analyses

All “case report forms” were the routinely administered MDHAQ and a physician-completed data sheet that included blood pressure, weight and all medications. These data were entered into a Microsoft Access database maintained on all patients seen at each visit in this setting and transferred to Stata V.9.2 (College Station, Texas, USA). The prednisone dose at baseline of all participants was compared descriptively with the initial dose of prednisone taken by patients at their first visit to this setting 1–15 years earlier.

Differences between treatment groups were evaluated using the Wilcoxon rank sum test for continuous variables or the Fisher exact test for categorical variables. The primary analysis and an intention-to-treat analysis of all randomised participants were conducted using the Fisher exact test to assess statistical significance. Differences between treatment groups with respect to changes from visit 1 to visit 5 or final visit for physical function score (0–10), pain visual analogue scale (VAS) score (0–10), patient global VAS score (0–10), RAPID3 composite score (0–30), fatigue VAS score (0–10), morning stiffness (minutes), weight, systolic/diastolic blood pressure, ESR and CRP levels and other laboratory tests were evaluated using the Wilcoxon rank sum test.

## Results

### Patient recruitment

Enrolment was conducted over 17 months from March 2005 to July 2006. Overall, 156 patients with RA were seen over this period. Although the trial was designed with liberal inclusion criteria and minimal exclusion criteria, only 37 of these 156 patients met the enrolment criteria and volunteered to participate. The reasons for non-participation included: 21 (13.5%) unwilling to discontinue taking prednisone, often noting previous efforts without success, at the advice of physicians, relatives and others; 21 (13.5%) clinically improving (RAPID3 lower by ⩾3 units) or with new RA therapies; 9 (5.8%) clinically declining (RAPID3 higher by ⩾3 units) with need for new therapies; 14 (9%) with severe fibromyalgia; 15 (9.6%) too far away for 3-monthly visits; 1 (0.6%) could not complete an English language questionnaire; 19 (12.2%) took a prednisone dose of >5 mg/day (all initiated by other physicians); 5 (3.2%) were not taking any prednisone; 4 (2.6%) with severe clinical status for whom the investigator regarded it as inappropriate clinically to discontinue prednisone; 3 (1.9%) pregnant or nursing; 5 (3.2%) with substantial comorbidities; and 2 (1.3%) with planned elective surgery. Thus, only 37 of the 156 patients with RA (23.7%) were eligible and volunteered to participate (table 2).

**Table 2:** Low-dose prednisone versus placebo in rheumatoid arthritis: enrolment results in 156 patients with rheumatoid arthritis

Total number of rheumatoid arthritis patients seen	156 (100%)
Improving or recently begun on new arthritis therapy	21 (13.5%)
Declining status	9 (5.8%)
Fibromyalgia as a major clinical problem	14 (9.0%)
Administrative: lives too far away	15 (9.6%)
Language barrier to completion of questionnaire	1 (0.6%)
Prednisone dose >5 mg/day	19 (12.2%)
Not taking any prednisone	5 (3.2%)
Refused to discontinue prednisone	21 (13.5%)
Comorbidity (AIDS, cancer, etc)	5 (3.2%)
Severe status (investigator not willing to risk discontinuing prednisone)	4 (2.6%)
Pregnant or nursing	3 (1.9%)
Surgery planned	2 (1.3%)
Enrolled	37 (23.7%)
Changed mind before randomisation	6 (3.8%)
Total randomised	31 (19.9%)

Of the 37 patients who agreed to participate in the trial, 6 (16.2%) reported that study prednisone was ineffective during the 12-week “equivalence” period compared with their usual prednisone (although the study tablets met US Food and Drug Administration (FDA) requirements) and declined to continue. Thus, 31 participants were randomised, 15 to the prednisone group and 16 to the placebo group.

### Participant enrolment in clinical trial

The participants randomised to prednisone and placebo did not differ significantly in age or any quantitative or laboratory measure (table 3). The mean prednisone dose at baseline was 2.9 mg/day and the median dose was 3 mg/day in both groups. Other treatments taken included methotrexate at doses between 5 and 25 mg/week by all but 2 participants; hydroxychloroquine by 10 participants (8 in combination with methotrexate), 5 in the prednisone group and 5 in the placebo group; leflunomide by 2 participants in the prednisone group; etanercept by 3 participants, 2 in the prednisone group and 1 in the placebo group; and adalimumab by 1 participant in the placebo group.

**Table 3:** Baseline mean level or percentage of participants randomised to placebo or prednisone

Variable	Placebo(N = 16)	Prednisone(N = 15)
Demographic variables		
Age (years)	50.1	53.3
Female (%)	62.5%	66.7%
Education (years)	13.5	15.1
Disease variables		
Disease duration (years)	4.4	8.1
Erythrocyte sedimentation rate (<28 mm/h)	18.9	14.6
C-reactive protein (0–10)	8.0	6.3
Questionnaire variables		
Physical function score (0–10)	1.02	1.36
Pain VAS score (0–10)	1.64	1.68
Global VAS score (0–10)	1.41	1.83
RAPID3 score (0–30)	4.07	4.87
Fatigue VAS score (0–10)	1.85	2.16
Morning stiffness (minutes)	38.8	21.7
Medication variables		
Methotrexate	93.8%	93.3%
Hydroxychloroquine	31.3%	33.3%
Leflunomide	0%	13.3%
Etanercept	6.3%	13.3%
Adalimumab	6.3%	0%

No differences between groups were statistically significant (p<0.05)

RAPID3, routine assessment of patient index data 3; VAS, visual analogue scale.

Among the 31 participants, 22 had a baseline prednisone dose of 3 mg/day, 5 of 4 mg/day, 3 of 2 mg/day and 1 of 1 mg/day (table 4). The initial dose of prednisone in these patients 1–15 years earlier had been 3 mg in 20 patients, 5 mg in 8 patients and 4 mg, 6 mg and 7.5 mg in each of 3 additional patients (table 4). The 15 participants randomised to receive prednisone included 1 with a baseline dose of 1 mg/day, 2 of 2 mg/day, 10 of 3 mg/day and 2 of 4 mg/day (table 5). Among the 16 participants randomised to receive placebo, 1 had a baseline dose of 2 mg/day, 12 of 3 mg/day and 3 of 4 mg/day (table 5).

**Table 4:** Initial prednisone dose at clinical setting 1–15 years before the clinical trial compared with prednisone dose at enrolment in clinical trial

Initial prednisone dose	Clinical trial dose				Total
	1 mg	2 mg	3 mg	4 mg	
3 mg	1	3	15	1	20
4 mg	–	–	1	–	1
5 mg	–	–	4	4	8
6 mg	–	–	1	–	1
7.5 mg	–	–	1	–	1
Total	1	3	22	5	31

**Table 5:** Clinical trial results in 31 participants randomised to prednisone or placebo following gradual withdrawal of prednisone, according to baseline prednisone dose

Study group	Clinical trial results	Baseline prednisone dose				Total
		1 mg	2 mg	3 mg	4 mg	
Prednisone	Number randomised	1	2	10	2	15
	Withdrew (lack of efficacy)	0	0	3	0	3*
	Completed trial	1	2	6	1	10*
	Withdrew (administrative)	0	0	1	1	2
Placebo	Number randomised	0	1	12	3	16
	Withdrew (lack of efficacy)	0	1	9	1	11*
	Completed trial	0	0	2	2	4*
	Withdrew (administrative)	0	0	1	0	1
Total		1	3	22	5	31

*For 28 participants who either completed the trial or withdrew because of lack of efficacy, p = 0.021 by Fisher exact test (prednisone vs placebo). For all 31 randomised participants, p = 0.032 by Fisher exact test (prednisone vs placebo).

### Withdrawal clinical trial results

Of the 15 participants randomised to the prednisone group, 2 were withdrawn for administrative reasons, one for an unexpected hysterectomy and the other for a recurrence of breast cancer. Of the 13 remaining participants in the prednisone group, 3 withdrew for lack of efficacy and 10 completed the 24-week “comparison” observation period (table 5).

Of the 16 participants randomised to the placebo group, 1 was withdrawn for administrative reasons; the patient had severe weight loss which was ultimately found to be based on depression, with discontinuation all medications. Of the 15 remaining participants in the placebo group, 11 withdrew for lack of efficacy and 4 completed the 24-week “comparison” observation period (table 5).

Differences between withdrawals in the prednisone group and the placebo group were statistically significant (p = 0.021, table 5). An intent-to-treat analysis of all randomised participants also indicated significant differences (p = 0.032, table 5).

Participants in the placebo group had higher median changes (indicating poorer status) with worsening scores for physical function, pain, patient global estimate, RAPID3 and fatigue. Participants in the prednisone group remained similar to baseline at the conclusion of the trial (table 6), although none of the differences were statistically significant compared with the placebo group (p>0.05). Furthermore, no significant differences were seen between the groups for changes in ESR or CRP levels.

**Table 6:** Median changes in measures from baseline to end point according to whether participant was randomised to placebo or prednisone

Measure	Placebo(N = 16)	Prednisone(N = 15)
Physical function score (0–10))	0.33	0.00
Pain VAS score (0–10)	0.50	0.10
Patient global VAS score (0–10)	0.65	0.00
RAPID3 composite score (0–30)	1.20	0.54
Fatigue VAS score (0–10)	0.45	0.00
Morning stiffness (minutes)	0.00	0.00
Erythrocyte sedimentation rate	−2.00	0.00
C-reactive protein	0.25	−0.30

No differences between groups were statistically significant (all p<0.05).

Negative sign indicates clinical improvement.

RAPID3, routine assessment of patient index data 3; VAS, visual analogue scale.

### Adverse events

No meaningful toxicities were reported by the participants in either group, as anticipated, since all participants had been taking stable doses of 1–4 mg/day prednisone before the trial, many for long periods. No significant changes in weight or blood pressure were seen within either group or between groups.

## Discussion

The results of this withdrawal clinical trial indicate that patients who were transferred from long-term prednisone doses of 1–4 mg/day to identical placebo tablets were significantly more likely to withdraw over a subsequent 6–9-month period than those who were randomised to prednisone. These results may appear surprising as most rheumatologists initiate (and often maintain) prednisone treatment at doses higher than 3 mg. By contrast, most participants in the clinical trial reported here had never taken prednisone at a dose higher than 3 mg, and the efficacy of this dose compared with placebo was documented in the trial.

This trial has many limitations. First, the number of participants is small, although a finding of statistically significant differences with only 31 participants may imply a robust treatment effect. Second, all participants were from one academic clinical practice and may not be representative of all patients with RA. A multicentre trial to improve generalisability of the results would be desirable. Third, a trial of *initiation* of prednisone 3 mg/day in patients who had never been treated previously with prednisone, rather than withdrawal from prednisone, might give more definitive information. However, a period of years would be required to accumulate a sufficient number of patients from one rheumatologist, and resources for performance of a multicentre trial have not been available. Fourth, the trial was conducted entirely in the course of usual clinic visits, without a study coordinator who might have added rigor to the results. However, the costs of this trial were substantially lower than in usual clinical trials and the primary outcome of withdrawal for lack of efficacy was accounted for in all 31 enrolled patients. It might be possible to conduct large simple clinical trials^31^ ^32^ in RA using only patient self-report measures and indices that include only these data. Self-report measures and indices distinguish active from control treatment as significantly as joint counts, laboratory tests or indices requiring these data in reported clinical trials of RA.^33^ ^34^

It was disappointing that only 37 of 156 consecutive patients with RA seen over 17 months were eligible and volunteered to participate, despite an effort to be inclusive, based on the observation that only a small fraction of patients with RA seen in this setting in the year 2000 were eligible for trials of anti-tumour necrosis factor α.^35^ Documentation of stable clinical status according to MDHAQ-RAPID3 data rather than non-quantitative clinical judgement may have introduced greater stringency in enrolment. In most clinical trials involving patients with “incomplete responses” to methotrexate, “stable” clinical status is based on clinical judgement rather than on quantitative data. This may explain why ACR20 responses in the range of 20–30% are seen in patients randomised to control treatment^36^ in “add-on” clinical trials of biological agents.^37^ A requirement for stable questionnaire scores over a 3-month period to document incomplete (or complete, stable or unstable) responses could reduce background responses in control arms of clinical trials of RA.

The reported trial may underestimate the treatment effects of prednisone, given that a primary reason for non-participation was a desire not to discontinue prednisone on the basis of failure of (often many) previous attempts. It is not clear why the six participants who withdrew before randomisation may have experienced lower efficacy with study prednisone than with their own prednisone. Although generic medications are required to meet chemical criteria for equivalence, anecdotal information suggests that some patients may vary in response to different brands of generic medications.

Neither efficacy nor safety of long-term low-dose prednisone can be established definitively from the results of this clinical trial. Long-term safety remains of concern. Higher mortality rates have been associated with the use of prednisone in an earlier cohort of patients seen by the senior author at Vanderbilt University^22^ and by others.^23^ ^38^ However, results of observational studies reporting adverse outcomes of glucocorticoids are confounded by indication, as patients with more severe clinical status are more likely to be treated with glucocorticoids. Furthermore, almost all patients in the previously reported studies had been treated with prednisone doses greater than 10 mg/day, many for extended periods.

Most participants in the present study never took doses of prednisone greater than 3 mg/day, with mean RAPID3 scores at baseline of <6 on a scale of 0–30, indicating low severity.^27^ Limited data are available concerning long-term mortality outcomes in such patients, although MHAQ physical function scores of <1.2 on a scale of 0–10 (0.6 on a scale of 0–3) are associated with favourable long-term mortality outcomes compared with all patients with RA.^39^ In one study of cardiovascular disease associated with long-term glucocorticoid use, patients whose dose was 5 mg showed no differences from control subjects.^40^ Large prospective studies as well as long-term observations of patients such as those in the present study, treated with prednisone only in doses of 5 mg/day or less, are needed to clarify possible effects of very low-dose prednisone on mortality.

We conclude that this clinical trial documents the efficacy of low-dose prednisone in patients with RA. Although not analysed in this study because of the short time frame, we have observed minimal long-term adverse events in patients who have taken daily prednisone for more than 10 years, sometimes up to 20 years. A multicentre long-term (2 years) “de novo” clinical trial of prednisone in new patients who have not had any prior glucocorticoid treatment would be of considerable value.
